# Impacts of the 2015 Heat Waves on Mortality in the Czech Republic—A Comparison with Previous Heat Waves

**DOI:** 10.3390/ijerph14121562

**Published:** 2017-12-13

**Authors:** Aleš Urban, Hana Hanzlíková, Jan Kyselý, Eva Plavcová

**Affiliations:** 1 Institute of Atmospheric Physics, Czech Academy of Sciences, Boční II 1401, 4 14131 Prague, Czech Republic; hanzlikova@ig.cas.cz (H.H.); kysely@ufa.cas.cz (J.K.); plavcova@ufa.cas.cz (E.P.); 2 Faculty of Science, Charles University, Albertov 6, 2 12843 Prague, Czech Republic; 3 Institute of Geophysics, Czech Academy of Sciences, Boční II 1401, 4 14131 Prague, Czech Republic; 4 Faculty of Environmental Sciences, Czech University of Life Sciences, Kamýcká 129, 6 16521 Prague, Czech Republic; 5 Global Change Research Centre, Czech Academy of Sciences, Bělidla 986, 60300 Brno, Czech Republic

**Keywords:** heat-related mortality, heat-wave, excess heat factor, Central Europe

## Abstract

This study aimed to assess the impacts of heat waves during the summer of 2015 on mortality in the Czech Republic and to compare them with those of heat waves back to the previous record-breaking summer of 1994. We analyzed daily natural-cause mortality across the country’s entire population. A mortality baseline was determined using generalized additive models adjusted for long-term trends, seasonal and weekly cycles, and identified heat waves. Mortality deviations from the baseline were calculated to quantify excess mortality during heat waves, defined as periods of at least three consecutive days with mean daily temperature higher than the 95th percentile of annual distribution. The summer of 2015 was record-breaking in the total duration of heat waves as well as their total heat load. Consequently, the impact of the major heat wave in 2015 on the increase in excess mortality relative to the baseline was greater than during the previous record-breaking heat wave in 1994 (265% vs. 240%). Excess mortality was comparable among the younger age group (0–64 years) and the elderly (65+ years) in the 1994 major heat wave while it was significantly larger among the elderly in 2015. The results suggest that the total heat load of a heat wave needs to be considered when assessing its impact on mortality, as the cumulative excess heat factor explains the magnitude of excess mortality during a heat wave better than other characteristics such as duration or average daily mean temperature during the heat wave. Comparison of the mortality impacts of the 2015 and 1994 major heat waves suggests that the recently reported decline in overall heat-related mortality in Central Europe has abated and simple extrapolation of the trend would lead to biased conclusions even for the near future. Further research is needed toward understanding the additional mitigation measures required to prevent heat-related mortality in the Czech Republic and elsewhere.

## 1. Introduction

Many studies have documented seasonal and meteorological effects on human mortality in various geographical areas [1,2,3]. In mid-latitudes, mortality shows a typical pattern with a peak occurring during the cold season and the lowest mortality rates in summer [4,5]. If mortality anomalies relative to the seasonal pattern are considered, however, periods of extremely high temperatures (heat waves) have a significant impact on mortality such as those in 1995 in the U.S. [6], 2003 in southwestern Europe [7], or 2010 in Russia [8]. As summer mean temperatures are expected to increase during the 21st century [9], the frequency and intensity of extreme heat waves are likely to be significantly greater in comparison to recent climate [10,11,12].

As a result of climate change, increased heat-related mortality is projected in the future [13,14,15]. Meanwhile, its compensation by reduced cold-related mortality has been questioned [9,16,17]. Despite increasing temperature in recent decades, a significantly reduced risk of heat-related mortality has been observed [18,19]. This has been documented in North America [20,21,22,23,24], Europe [23,25,26,27,28], as well as Asia and Australia [23,29,30]. The magnitude and significance of heat-related mortality change may differ, however, according to region, length of the examined period, number and timing of heat waves, and the methodology used [23,26,31,32,33]. Although many studies have assessed temporal changes in relative risk of increased mortality per unit temperature rise [21,24], much less has been done to compare effects of heat events on mortality with respect to their meteorological and spatial-temporal characteristics [34,35,36].

Physiological (acclimatization), behavioural (clothing, working time), and technological (urban planning, building design, warning systems) adaptations have been often hypothesized as the reasons for declining vulnerability to extreme temperatures [21,24,25,26]. Although increased use of air conditioning has been considered one of the main causes of reduced susceptibility to heat [19,24,37], larger temporal declines in heat-related mortality were not significantly linked to larger increases in air conditioning prevalence when different cities and regions were compared [21,30]. Limited understanding as to which specific mechanisms and adaptation measures have caused the decrease in heat-related mortality [18,19,38] brings uncertainty to projections for the effect of future climate change on temperature-related mortality [13,14,15,39,40]. Therefore, further research is required to improve the effectiveness of such planned adaptation measures as heat warning systems [38,41]. This includes seeking to better understand heat-wave characteristics that affect human health.

In 1994, Central Europe experienced its hottest summer since at least the 1950s [42]. An exceptional impact on excess mortality was accordingly documented in several countries, including the Czech Republic [43,44], Germany [45], Poland [46], and The Netherlands [47]. Since 1994, several notable heat waves have occurred in the Central European area, including those in 2003 [44], 2006 [48,49], and 2013 [50]. Globally the hottest year to that date was 2015 [51], and Central European countries experienced intense, record-breaking heat waves [52,53]. In Switzerland, the summer of 2015 had as great impact on excess mortality as did the climatologically comparable summer of 2003 [54]. In England, on the other hand, the impact of the 2013 heat wave was considerably less than seen in 2003 and 2006 even though the temperature magnitude of the heat waves was comparable [55]. These two examples demonstrate that the impact of a given heat wave on mortality is affected by more factors than simple temperature characteristics. Duration and intensity of a heat stress period [25,36], timing of a heat wave within a season [44,56,57], air quality in a given area [49], and/or the magnitude of mortality in the previous winter due to influenza epidemics [44,58] can substantially influence the overall impact. Additionally, the structure of a given population, including its demographics and socioeconomic status, can significantly affect the impact of heat on mortality in a specific region [59,60,61,62]. Although the impact of hot weather on mortality is generally largest in urban environments [63], it may be significant also in rural areas [62,64].

Several studies have evaluated mean effects of hot spells on mortality [65] and morbidity [66] in the Czech Republic, as well as their spatial differences [67], using data up to 2009. Hůnová et al. [49] examined the effect of heat waves in 2003 and 2006 on mortality in Prague, but to date the impacts of the heat waves since 2010 have not been evaluated. This study aims to assess the impact of the 2015 heat waves on excess natural-cause mortality in the Czech Republic and to compare it with previous heat waves back to 1994 while focusing on the previously record-breaking summer of 1994. Meteorological characteristics of heat waves, considering their intensity, duration, and temperature change with respect to the previous conditions, are compared and linked to the magnitude of heat-related mortality during heat waves. The decreasing trend in heat-related mortality documented by previous studies is evaluated in the context of changes in impacts of heat waves, and demographic and meteorological factors modifying the observed impacts are discussed.

## 2. Data and Methods

### 2.1. Mortality Data

Daily natural-cause mortality (A00-R99 according to the International Classification of Diseases, version 10 (ICD-10)) was analysed in the Czech Republic during 1994–2015 (population about 10.5 million inhabitants as of 2015, having changed only little since 1994). The data were collected and processed by the Czech Statistical Office and the Institute of Health Information and Statistics of the Czech Republic. The data were stratified by gender and age into four subgroups—a total of 2,253,788 deaths were recorded in the national registry during the examined period, out of which 1,110,755 were males, 1,143,033 females, 486,789 younger than 65 years (0–64 years), and 1,766,999 deceased were 65 years and older (65+ years). As the demographic structure of the population has shifted toward a higher proportion of the elderly during the examined period (see Supplementary Materials Figure S1), we calculated daily mortality rates (per 10,000,000 inhabitants) in order to account for these changes. We applied the direct standardization procedure [67] using the mid-year population in each year and the standard WHO European population as a reference [68].

### 2.2. Meteorological Data

**Figure 1:**
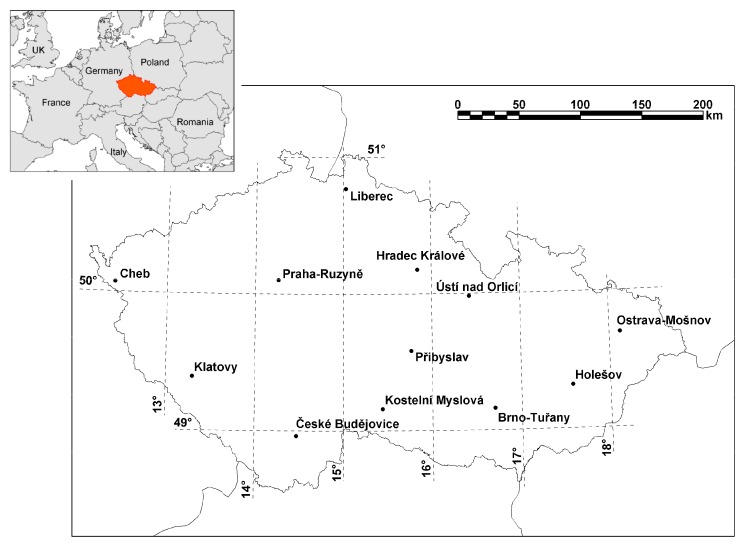
Location of the Czech Republic and meteorological stations used to calculate mean temperature (black dots).

Daily air temperature was calculated as the mean from 12 high-quality meteorological stations operated by the Czech Hydrometeorological Institute, representing the spatial population distribution in the Czech Republic (Figure 1), and used as a proxy variable of thermal conditions. While some studies have shown a theoretical advantage of indices combining multiple variables in order to assess human thermal comfort [69] as well as their better performance on mortality data [70,71], the differences between various temperature measures in evaluating heat’s impact on mortality are generally insignificant [36,72,73,74].

### 2.3. Definition of a Heat Wave

To select only those heat waves affecting the majority of the area, we used one temperature time series calculated from the 12 stations distributed across the whole area. Heat waves are defined as periods of at least 3 days with daily mean temperature (Tmean) reaching or exceeding the 95th percentile (21.2 °C) of the annual distribution (estimated from data over 1994–2015), with at least 1 day exceeding the 98th percentile (23.2 °C). Although there is no universally accepted heat wave definition in public health studies, the 95th percentile of temperature distribution is generally understood as a threshold of a heat wave according to the WHO and WMO Guidance on Warning-System Development [75]. The second threshold (the 98th percentile) is employed in order to ensure that a heat wave involves at least one day with very high temperature; definitions of heat waves with two thresholds are relatively frequent in biometeorological and climatological studies [44,47,76].

### 2.4. Excess Heat Evaluation

Koppe and Jendritzky [56] pointed out the importance of short-term (within-season) acclimatization to heat for the overall impact of a heat wave on human mortality, and accordingly they introduced the HeRATE approach to heat-related mortality assessment. A similar method by Nairn and Fawcett [77] employs the excess heat factor (EHF) for evaluating the heat wave intensity with respect to previous thermal conditions. EHF consists of two excess heat indices (EHIs): the significance index (EHIsig), a measure of heat wave intensity, and the acclimatization index (EHIaccl), taking into account short-term acclimatization to ambient temperature over the recent past. Daily EHIsig and EHIaccl were calculated using Equations (1) and (2), respectively:

EHIsig*_i_* = (T*_i_* + T*_i_*_–1_ + T*_i_*_–2_)/3 − T^95^, and (1)

EHIaccl_i_ = (T*_i_* + T*_i_*_–1_ + T*_i_*_–2_)/3 − (T*_i_*_–3_ + … + T*_i_*_–32_)/30, (2)

where T*_i_* denotes daily mean temperature on day *i*, T^95^ represents the 95th percentile of daily mean temperature, EHIsig*_i_* is defined as the exceedance of the previous three-day mean temperature (starting on day *i*) above T^95^, and EHIaccl*_i_* on day *i* is calculated as the difference between the three-day mean temperature and a mean of the prior 30 days. It should be noted that we slightly modified the original Nairn and Fawcett’s (2015) equations by calculating the three-day mean temperature over days *i* to *i* − 2 instead of days *i* to *i* + 2, since human mortality on day *i* can be affected by thermal conditions on day *i* or previous days, but not on upcoming days. Consequently, daily EHF was calculated using Equation (3):

EHF*_i_* = max(0,EHIsig*_i_*) × max(1,EHIaccl*_i_*) (3)

Finally, values of EHIsig, EHIaccl, and EHF on all days of a heat wave were summed, in order to evaluate its total heat load.

### 2.5. Statistical Analyses

#### 2.5.1. Excess Mortality Calculation

To assess the mortality increase during heat waves, daily baseline for natural-cause mortality was determined using generalized additive models (“mgcv” package in R (version 2.15.2) [78]). Specifically, we employed Equation (4):

Log[*E*(*M*)] = *α* + *s*(*time*; *df* = 6 × *n*(*y*)) + *factor*(*dow*) + *factor*(*hwd*), (4)

where *E(M)* is the predicted daily mortality, *α* is the model intercept, *time* is a variable to account for long-term trends and seasonality, *n*(*y*) is the number of years in the time series, *s*() represents thin plate smoothing spline [78], and *factor* variables define categorical variables for days of week (*dow*) and heat-wave days (*hwd*). A smoothing spline with six degrees of freedom (*df*) per year (total *df* = 132) was applied after sensitivity tests evaluated by a combination of procedures, including partial autocorrelation plots, models’ unbiased risk estimator criterion [78], percentage of deviation explained, and visual examination of model-predicted baseline plots. The final number of *df* used was also in accordance with an estimate (137 *df*) made via generalized cross-validation using the “bruto” function in the “mda” R package (version 0.4-9 [79]) according to Peng and Dominici [80]. The weekly cycle in mortality data, with maximum on Monday and minimum on Sunday was adjusted by the categorical variable *dow*. Finally, the mortality baseline was adjusted by a categorical variable representing days of heat waves identified in this study (*hwd*). The *hwd* variable reaches values 0/1, where 1 is used for the heat wave days. The coefficient of the *hwd* variable was subtracted from the model-predicted baseline on heat-wave days and therefore the excess mortality during the heat waves was calculated with respect to the baseline adjusted for the effect of heat waves [55].

Daily numbers of deaths per standardized 10 million inhabitants and their relative deviations from the mortality baseline (excess mortality) are shown in Figure S2. Cumulative excess mortality per standardized 10 million inhabitants during the identified heat waves was calculated for the population as a whole and individually for population groups showing usually different response to heat: males and females, and age groups younger and older than 65 years (0–64 and 65+ years, respectively). The significance of the mortality excess was evaluated by 95% confidence intervals of the cumulative excess mortality, calculated as the lower and upper limit factors for a Poisson-distributed variable according to Schoenberg [81].

#### 2.5.2. Associations between Heat Waves and Excess Mortality

Characteristics of heat waves such as average daily mean temperature (average Tmean), intensity (EHIsig), duration, and timing within a year have been documented as important factors modifying the eventual impact on mortality [34,36]. Using the cumulative excess heat factor (∑EHF) allows us to consider all these important meteorological components defining the total heat load of a heat wave together—its intensity represented by the temperature anomaly during the heat wave (EHIsig), timing within a season represented by the temperature change with respect to the previous conditions (EHIaccl), and duration (∑). To evaluate associations between heat waves and mortality with respect to the baseline mortality in a given year, the sum of relative mortality deviations (∑RMD) during a heat wave was calculated and linked to the above mentioned heat wave characteristics, using linear regression models.

#### 2.5.3. Mortality Displacement

Although heat waves are defined by the temperature thresholds presented above, the actual periods of heat stress are likely to be slightly longer (e.g., a hot period may have started to develop several days before a heat wave) and the total effect of a heat wave may be slightly greater than that of the identified heat wave period. On the other hand, the effect of a short-term mortality displacement (harvesting) after the end of a heat wave may substantially reduce the net effect of a heat wave on excess mortality [82,83]. To evaluate the magnitude of these two effects, we defined a so-called “extended heat wave period” (EHP) [43] with several days of positive mortality deviations before a heat wave and several days with negative mortality deviations after a heat wave. A three-day centered moving average of mortality deviations was calculated to evaluate the mortality displacement. The beginning of EHP was delimited as the first three-day period with a positive average mortality deviation before a heat wave while the end of EHP was defined as the last three-day period with a negative average mortality deviation after a heat wave [43]. A positive phase of EHP lasted till the central day of the last three-day period with positive averaged mortality deviations and the rest of EHP was defined as a negative phase. Multiple heat events separated by days without a drop of the three-day average of mortality deviations below zero were considered as a single EHP. The final magnitude of the mortality displacement was calculated as the absolute value of the ratio of the sum of the three-day-averaged mortality deviations during the negative phase of EHP to the sum of the three-day-averaged mortality deviations during the positive phase.

## 3. Results

### 3.1. General Characteristics of Heat Waves

A total of 49 heat waves were identified between 1994 and 2015 in the Czech Republic, with maximum of four heat waves per year (Table 1). Although we did not strictly specify the season of our analyses, all identified heat waves (except one in May 2005) occurred in summer (June–August). Most of the heat waves were associated with positive cumulative mortality deviation and the positive anomalies were significantly (*p* = 0.05) different from the mortality baseline in 40 events (see Supplementary Materials Tables S1 and S2A,B). The mean relative rise in natural-cause mortality during the heat waves was 10.7% per day, while the maximum daily excess mortality was greater than 30% on the hottest days. Differences in the mean impact of heat waves were observed between population groups. While the mean relative mortality deviation during heat waves was not significantly (*t*-test at *p* = 0.05) different between women and men (11.7% vs. 10.1%, respectively), it was significantly larger in the elderly (65+ years) than the younger (0–64 years) age group (11.6% vs. 8.3%, respectively).

**Figure 2:**
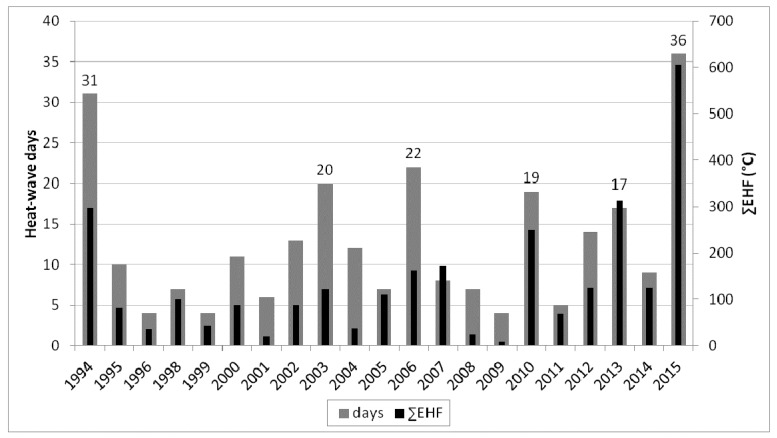
Total duration of heat waves in each year during 1994–2015 in the Czech Republic, and their total heat load according to the cumulative excess heat factor (∑EHF).

2015 was the record-breaking year during the examined period regarding the total duration of heat waves (36 days) as well as their cumulative excess heat factor (∑EHF = 606 °C; Figure 2). Table 1 shows meteorological characteristics of heat waves in six years with the total heat wave duration longer than 15 days. Although the longest heat wave during the examined period occurred in 1994 (18 days; 22 July–8 August; denoted 1994_4), the heat wave 2015_3, lasting 13 days (3–15 August), was the hottest in the study period according to the average daily mean temperature (avgTmean = 26.0 °C, compared to 24.1 °C in 1994_4) as well as ∑EHF (292.4 °C and 176.6 °C, respectively).

### 3.2. Effects of the Major Heat Waves on Excess Mortality

The two persistent heat waves—1994_4 and 2015_3 (hereafter termed *major* heat waves)—were remarkable not only because of their meteorological characteristics, but also due to their impact on excess mortality, which was substantially larger than during any other heat wave (Table 2 and Table S2A,B). The 1994_4 major heat wave was associated with 1197 excess deaths (95% confidence interval (95% CI: 1002–1397) per standardized 10 million inhabitants, in comparison to 847 (95% CI: 711–988) deaths per 10 million inhabitants during 2015_3. However, these values represented 240% and 265% cumulative relative mortality deviation (∑RMD), respectively, thus indicating that the impact on mortality relative to the mortality baseline in the given year was larger in 2015. A substantial difference in the impact of the two major heat waves was observed between the two age groups. While ∑RMD was comparable among the younger and the elderly age groups (254% and 234%, respectively) in the 1994_4 heat wave, it was significantly larger (*p* = 0.05) among the elderly in 2015_3 (132% and 313%, respectively).

**Figure 3:**
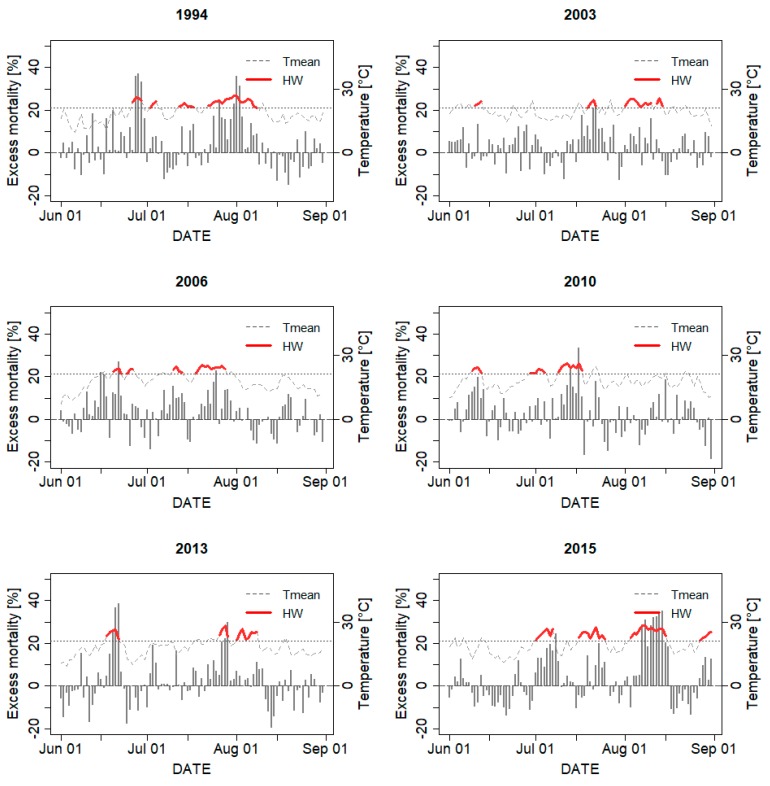
Relative excess mortality during summers (June–August) in six years with the total heat wave (HW) duration greater than 15 days. Tmean indicates daily mean temperature for the Czech Republic. A horizontal dotted line shows the heat wave threshold temperature—95th percentile of the annual daily mean temperature distribution during 1994–2015.

Although the fourth heat wave in 2006 (18–28 July; 2006_4) was the third longest heat wave during the examined period (11 days) and avgTmean was equal to 1994_4 (24.1 °C), its impact on ∑RMD (93%) ranked as the 11th largest among all heat waves. Similarly, the longest heat wave in 2003 had a relatively small impact on mortality (∑RMD = 60%), despite its 10-day duration (1–10 August; 2003_3). In both cases, the values of ∑EHF (72.7 °C in 2003_3 and 99.7 °C in 2006_4, respectively) were much lower than during the major heat waves (1994_4 and 2015_3). On the contrary, other heat waves, such as 1994_1, 2007_1 (Tables S1 and S2B), 2013_1, and 2015_1 had quite a strong impact on excess mortality despite their relatively short duration (4, 8, 5, and 7 days, respectively). These heat waves occurred as the first heat waves in a given season, accompanied by a substantial increase of temperature with respect to previous conditions (Table 1 and Figure 3). Therefore, their total heat load was also relatively large (∑EHF = 99.4, 171.3, 158.7, and 143.1 °C, respectively).

### 3.3. Associations between Heat Wave Characteristics and Excess Mortality

**Figure 4:**
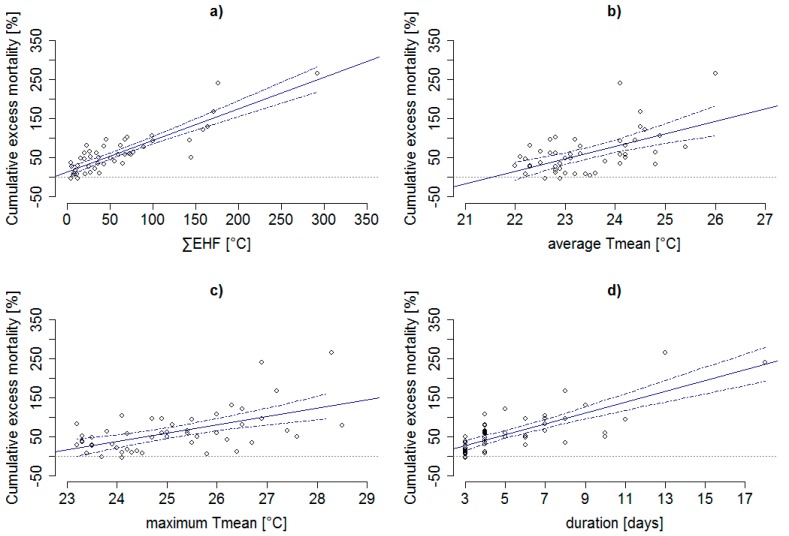
Relationship between cumulative excess mortality for the whole population and (**a**) cumulative excess heat factor (∑EHF), (**b**) average daily mean temperature (average Tmean), (**c**) maximum daily mean temperature (maximum Tmean), and (**d**) duration of heat waves, during 1994–2015 in the Czech Republic. Dash-dotted lines indicate 95% confidence interval of the regression line. Slopes of the fitted regression lines are presented in Table 3.

The above presented results highlight the importance of considering the total heat load of a heat wave when evaluating its impact on human health. This finding was also supported by the linear regression analysis performed among all 49 heat waves. As shown in Figure 4, increasing ∑EHF during a heat wave was significantly linked to increasing ∑RMD (R^2^ = 0.75). The ∑RMD of a heat wave depended more strongly on ∑EHF than other indicators of heat wave intensity such as average (avgTmean, R^2^ = 0.31) and maximum daily mean temperature (maxTmean, R^2^ = 0.31) during a heat wave, as well as its duration (R^2^ = 0.59). Table 3 shows that relationships between ∑RMD and independent variables were generally stronger among the elderly (65+ years) than the younger (0–64 years) age group.

### 3.4. Mortality Displacement

Generally, the order of heat waves with the largest impact on mortality was maintained also if the mortality displacement effect during the extended heat wave period (EHP) was considered (Table 2). The net cumulative mortality deviation was positive for most EHPs (see Table S3). In several cases, the net cumulative mortality deviation during EHPs was larger than during the heat waves alone due to an extraordinarily long period of consecutive positive daily excess mortality preceding or following the heat wave. The proportion of the harvesting effect on excess mortality during the positive phase of EHPs was estimated to be on average 23% during the most extreme heat waves (with ∑EHF ≥ 100 °C), while it was larger, on average 54%, during heat waves with ∑EHF < 100 °C. The magnitude of the mortality displacement effect varied substantially between individual cases, however. While the magnitude of the mortality displacement exceeded 30% during EHPs of the intense but relatively short heat waves in 2007 (2007_1) and 2013 (2013_1) (38% and 37%, respectively), comparable heat waves in 2010 (2010_3) and 2015 (2015_1) revealed a substantially lower harvesting effect (5% and 10%, respectively). In the latter two cases, the harvesting effect of the heat waves was weakened by a subsequent hot period occurring shortly thereafter (Figure 3). This was true especially in 2015, when the 2015_1 heat wave (7 days; ∑EHF = 143.1 °C) was followed by the even more severe 2015_2 heat wave (10 days; ∑EHF = 145.1 °C), but the impact of the second heat wave on excess mortality was substantially weaker. When only the two major heat waves (1994_4 and 2015_3) are compared, the magnitude of the mortality displacement was slightly larger in 1994 (27%) than in 2015 (22%).

## 4. Discussion

We analyzed effects of heat waves on natural-cause mortality in the Czech Republic during summer 2015 and compared them with the effects of previous heat waves back to 1994. Two major heat waves, occurring on 22 July–8 August 1994 and 3–15 August 2015, respectively, were exceptional in both the climatological extremity and their impact on excess mortality. Although the excess mortality (deaths per standardized 10 million inhabitants) was higher in 1994, the increase in mortality relative to the baseline in a given year was larger in 2015. These findings suggest that the health response to the major heat-stress events has not improved significantly during the examined period and that the total heat load of a heat wave, given by its intensity, duration, and temperature change with respect to the previous conditions, constitutes one of the main factors affecting the magnitude of the mortality increase.

### 4.1. Effect of the Total Heat Load

Studies reporting significant decrease in heat-related mortality over time have mostly assessed temporal changes in relative risk of increased mortality per unit of temperature increase, aggregating all days above a certain threshold [21,24]. This approach may improve our knowledge of temperature–mortality relationships in general, but it does not tell much about effects of individual heat events with respect to their meteorological and spatial-temporal characteristics [34,35,36]. The extraordinary impact of summer 2015 on heat-related mortality has been already documented in several Central European countries [54,77]. The relative mortality increase during the 2015 major heat wave in the Czech Republic was larger than in 1994, even though the 2015 event was shorter by five days. When comparing the overall magnitude of the two major heat waves, however, the 2015 one was the most extreme during the examined period as measured by average daily mean temperature during the heat wave (avgTmean), the cumulative temperature anomaly above the 95th percentile threshold (∑EHIsig), cumulative temperature increase with respect to the previous conditions (∑EHIaccl), as well as cumulative excess heat factor (∑EHF). These findings are in accordance with those of Hoy et al. [53], who have reported summer 2015 in the Central European region as record-breaking since the beginning of the 20th century in terms of several heat-wave characteristics.

On the other hand, while the summer of 2003 (June–August) was the second warmest in the Czech Republic during the examined period (according to mean temperature), temperature increases during heat waves were generally small compared to the non-heat wave conditions, thus resulting in low cumulative excess heat factor of the heat waves in 2003. Therefore, despite its length comparable to the 2015 major heat wave, the total heat load of the August 2003 heat wave was substantially lower. The importance of the total heat load of a heat wave (considered as a function of its absolute temperature anomaly, temperature change, and duration [34,36]) in the final effect on heat-related mortality was demonstrated in this study by the strong correlation between the cumulative relative mortality deviation (∑RMD) and ∑EHF during a heat wave. The difference between the low total heat load during the 2003 heat wave in Central Europe demonstrated here as well as in other studies [31,42,45,77] and, on the other hand, its strong intensity in Western Europe [50,52], contributed significantly to the spatial differences in the mortality response across Europe [44].

Despite the declared importance of the total heat load in the final impact of a heat wave on excess mortality, heat waves with comparable ∑EHF in our study had in some cases substantially different impact on ∑RMD. These variations were related mainly to different conditions preceding the heat waves. For example, while in 2007 a mild summer without obvious temperature extremes prevailed in Central Europe prior to the heat wave with the third largest ∑RMD during the examined period, a comparably intense heat wave in 2010 (2010_3) had a weaker impact on excess mortality as it was preceded by two other heat waves earlier in the season. On the other hand, the cumulative excess mortality during both heat waves was comparable when the extended heat wave periods (EHPs) including the harvesting effect were considered. This finding emphasizes limitations of the regression analyses of associations between ∑RMD and heat wave characteristics, assuming that all heat-related excess deaths occurred only during the identified heat waves. On the other hand, it is also not very likely that all excess deaths that occurred during EHPs were associated to the heat stress. Although we attempted to estimate mortality displacement during individual EHPs, the resulting values have to be perceived very carefully, since quantifying the real proportion of deaths that would have occurred regardless of the heat wave is still not a completely resolved issue [82,83].

In addition, the study did not take into account other potential confounding variables or effect modifiers, such as air pollution [84] and mortality in previous winters due to influenza epidemics [85,86]. As air pollution concentrations vary considerably across space and causal relationships among temperature, air pollution, and human health are associated with large uncertainty [87], we did not consider the effect of air pollution in this study. Possible effects of the influenza epidemic in the winter season 2002/2003 on reduced excess mortality during the following summer have been discussed by Kyselý and Kříž [44]. A study from Slovakia [76] suggests that the impact of summer 2015 on excess mortality could have been even stronger, if no influenza epidemic occurred earlier in the year.

### 4.2. Changes in Population Structure and Socioeconomic Factors

In addition to meteorological factors, demographic changes over time may significantly modify the effect of heat. Age-standardization of mortality is commonly applied in epidemiological studies in order to adjust the time series for changes in population structure over time and space [68]. Due to age-standardization, lower heat wave-related mortality (deaths per 10 million inhabitants) was estimated in 2015 than in 1994. However, when the relative mortality deviations from the baseline were considered, the impact of the 2015 major heat wave was even greater than that of the 1994 heat wave. This finding seems to be in contradiction with Kyselý and Plavcová [26], who found a significantly decreasing trend in relative excess heat-related mortality in the Czech Republic over 1986–2009. The significant decrease was related primarily to substantial socioeconomic development, improved quality of life (including more efficient health care and lifestyle changes), and significantly decreased environmental deprivation due to rapid transition from a developing to developed economy in the Czech Republic (and other Central European countries) in the 1990s, following the so-called “Velvet Revolution” and end of the communist regime in 1989 [26]. Socioeconomic and lifestyle changes were much less pronounced in the most recent past, which may have contributed to abating the trend in heat-related mortality. A third factor contributing to significantly decreasing heat-related mortality over 1986–2009 and reported by Kyselý and Plavcová [26] might have been essentially the absence of a major heat wave with an impact on mortality comparable to that of the 1994 event in the second half of their examined period. Ha and Kim [32], for example, excluded summer 1994 with an unprecedented heat wave in South Korea from their analysis, thus resulting in predominantly insignificant changes in temperature-related mortality over time (1993–2009).

Significant decreases in heat-related mortality risk during recent decades have been observed also in the U.S. [20] and other developed regions of the world [23,25,28,30]. These have been associated mainly with improved health care, better public awareness of heat-related risks, and increased prevalence of air conditioning [20,24,88]. The decline has abated since the mid-1990s in the U.S., however, and a further substantial decrease may be unlikely, as the air conditioning availability is already near its saturation in some regions [24,88,89]. The results of our study suggest that a similar abatement of this favourable development may be observed or expected in other regions, too, albeit for different reasons (air conditioning is much less widespread in Central Europe), and that major or unprecedented heat waves may substantially influence the estimated trends. As their frequency, intensity, and duration are projected to increase in a warmer climate, a simple extrapolation of observed trends in heat-related mortality would lead to biased conclusions even for the near future.

Together with the favourable socioeconomic development over recent decades, the demographic structure of the population has shifted toward a higher proportion of the elderly during the same period. While in 1994, 13% of the Czech population was older than 65 years of age, this figure was already 18% in 2015 [90]. Our results showed that the relative increase in mortality was comparable between the younger age group (0–64 years) and the elderly (65+ years) during the 1994 major heat wave, while it was substantially larger among the elderly in 2015. These findings suggest that the 0–64 years age group has become less vulnerable to heat stress over time, while the total impact of the 2015 major heat wave was comparable with that of the 1994 heat wave due to increased proportion of the elderly (65+ years) among the affected population. An analysis at a finer detail of age groups would be needed to understand our findings in the context of changing population structure and increasing life expectancy. However, as the elderly with chronic cardiovascular and respiratory diseases constitute the population group at the highest risk of heat-related death [57,65,66] and their proportion in the total population has been increasing over time, they should be in the particular interest when developing new adaptation measures reducing impacts of the extreme heat on public health. In order to quantify the effects of population ageing on changes in the heat-vulnerability properly, a measure such as years of life lost [16]—considering the life expectancy at the time of death—should be analyzed in follow-up research.

### 4.3. Heat-and-Health Warning Systems

Heat-and-health warning systems (HHWSs) have been often presented as an effective tool of heat-related mortality alleviation [9,41,91]. Reduced impact of heat waves on mortality has been documented in southwestern European countries after the 2003 heat wave, when local HHWSs were set up [55,92,93]. On the other hand, a study by de’Donato et al. [33] comparing changes in heat-related mortality risk between two seven-year periods before and after 2003 showed that the beneficial effect of HHWSs has not been consistent across Europe. In spite of many methodological similarities among HHWSs in various countries, significant differences in key characteristics of the plans exist [94], especially regarding steps following the heat alert issuance. Therefore, it is necessary to evaluate the effectiveness of these systems in order to understand which actions actually increase resilience of populations [9,38,41,94].

The Czech Republic does not so far have an HHWS based on temperature–health relationships and including steps to follow after an alert’s issuance [94]. As demonstrated by recent studies [33,95], North and Central European cities may be particularly vulnerable to increasing intensity and frequency of heat waves in the near future. The findings of this study suggest that meteorological characteristics of a predicted heat period should be considered within the context of the previous temperature and mortality development when its possible impact on excess mortality is estimated [44,55]. Otherwise, the heat wave’s severity in relation to impacts may be misinterpreted. Excess heat factor [77] or a similar method considering the total heat load of a heat wave with respect to previous conditions may be one of the ways to improve HHWSs (as already documented by [96]), as better understanding of weather events is essential for properly predicting the possible human health impact [24,97].

For better understanding of associations between weather events and human health when developing an effective HHWS, the WMO/WHO guideline [75] recommends consideration of location-specific relationships between ambient temperature and mortality. Therefore, it needs to be acknowledged that associations between heat waves and excess mortality found in this study for the nation-wide population may not necessarily represent associations existing in individual cities and regions. As previous study has shown [67], however, modelling temperature-mortality relationships in relatively small cities and regions is difficult due to small sample sizes that do not bring robust results. Moreover, as the aim of this study was to compare the impact of the extraordinary summer 2015 on excess mortality with the previous important heat waves back to 1994 and not to define a HHWS methodology for a specific city, we followed the methodological approach applied in previous studies assessing the impact of heat waves on mortality in the Czech Republic and its changes over time [26,43,44].

## 5. Conclusions

Impacts of heat waves in summer of 2015 on natural-cause mortality in the Czech Republic were evaluated and compared with those of previous heat waves back to the record-breaking summer of 1994. The summer 2015 exceeded the 1994 one in both the intensity of heat waves and magnitude of heat-related mortality. Despite the smaller excess mortality associated with the major heat wave in 2015 in comparison to 1994, the relative increase from the baseline was larger in 2015. The effect of the major 2015 heat wave was more pronounced in particular due to stronger impact on excess mortality among the elderly (65+ years) than the younger age group (0–64 years) in 2015 compared to 1994. The results suggest that the cumulative excess heat factor of a heat wave, considered as a function of absolute temperature anomaly during a heat wave, heat wave duration, and temperature increase relative to the previous conditions, explains better the magnitude of excess mortality during a heat wave than other characteristics such as duration and average daily mean temperature during the heat wave alone. Further research is needed to improve the effectiveness of measures to prevent heat-related mortality while considering changes in the intensity, duration, and frequency of heat waves due to ongoing climate change as well as changes in the demographic, epidemiological, and socioeconomic structure of the population over space and time. The comparison of mortality impacts of the 2015 and 1994 major heat waves suggests that the recently reported decline in overall heat-related mortality in Central Europe has abated and a simple extrapolation of the trend would lead to biased conclusions even for the near future.

## Figures and Tables

**Table 1:** Characteristics of heat waves within six years with the longest total heat-wave duration during 1994–2015 in the Czech Republic.

Heat Wave	Start	End	Days	avgTmean (°C)	maxTmean (°C)	∑EHIsig (°C)	∑EHIaccl (°C)	∑EHF (°C)
**1994_1**	26 June	29 June	4	24.9	26.0	9.1	34.9	99.4
**_2**	2 July	4 July	3	22.8	24.2	0.7	14.2	10.0
**_3**	12 July	17 July	6	22.0	23.2	2.8	15.4	12.2
**_4**	22 July	8 August	18	24.1	26.9	51.5	49.3	176.6
**2003_1**	10 June	12 June	3	23.2	24.3	3.6	16.9	21.1
**_2**	19 July	22 July	4	22.8	25.0	3.1	15.2	20.0
**_3**	1 August	10 August	10	23.3	25.4	17.1	30.7	72.7
**_4**	12 August	14 August	3	23.5	25.8	5.1	4.4	8.2
**2006_1**	19 June	22 June	4	22.7	23.8	3.3	32.2	34.4
**_2**	24 June	26 June	3	22.9	23.7	1.0	19.2	12.7
**_3**	10 July	13 July	4	23.1	24.7	6.2	9.6	15.9
**_4**	18 July	28 July	11	24.1	25.5	26.5	30.4	99.7
**2010_1**	9 June	12 June	4	23.1	24.2	5.8	38.3	59.9
**_2**	29 June	4 July	6	22.3	23.5	3.7	29.4	25.4
**_3**	9 July	17 July	9	24.5	26.3	24.1	48.3	163.7
**2013_1**	17 June	21 June	5	24.6	26.5	13.5	52.8	158.7
**_2**	26 July	29 July	4	25.4	28.5	13.2	24.5	90.0
**_3**	1 August	8 August	8	24.1	26.7	17.7	22.5	65.2
**2015_1**	1 July	7 July	7	24.4	26.9	16.0	46.3	143.1
**_2**	16 July	25 July	10	24.2	27.6	26.0	48.2	145.1
**_3**	3 August	15 August	13	26.0	28.3	57.6	55.3	292.4
**_4**	27 August	1 September	6	22.5	25.4	8.1	4.9	26.2

**Table 2:** Impact of heat waves on mortality for the whole population, and the younger (0–64 years) and the elderly (65+ years) age groups, within six years with the longest total heat-wave duration during 1994–2015. The variables represent sum of excess deaths per standardized 10,000,000 inhabitants (excess mortality) and its 95% confidence intervals (CI), mean relative mortality deviation (ØRMD), and cumulative relative mortality deviation (∑RMD) during heat waves. Displaced mortality represents the absolute value of the ratio of the sum of three-day-averaged mortality deviations during the negative phase of the extended heat wave period (EHP) to the sum of three-day-averaged mortality deviations during the positive phase (see Section 2.5.3). --- indicates that the heat wave was considered as a single EHP together with the following one. * denotes statistically significant excess mortality.

Heat Wave	Whole Population				0–64 Years			65+ Years		
	Excess Mortality (*n*)	ØRMD (%)	∑RMD (%)	Displaced Mortality (%)	Excess Mortality (*n*)	ØRMD (%)	∑RMD (%)	Excess Mortality (*n*)	ØRMD (%)	∑RMD (%)
**1994_1**	552 (455; 654) *	26.8	107.2	---	63 (17; 115) *	10.9	43.7	487 (403; 577) *	32.7	130.8
**_2**	89 (12; 170) *	5.8	17.3	18.4	53 (12; 98) *	12.3	36.9	36 (−28; 105)	3.3	9.8
**_3**	144 (36; 257) *	4.8	28.9	28.3	21 (−35; 80)	2.4	14.3	123 (32; 220) *	5.7	34.1
**_4**	1197 (1002; 1397) *	13.4	240.3	26.6	352 (250; 459) *	14.1	254.4	844 (678; 1014) *	13.0	234.1
**2003_1**	40 (−31; 115)	3.0	8.9	21.8	19 (−17; 59)	5.4	16.1	21 (−40; 85)	2.0	6.1
**_2**	265 (181; 355) *	15.5	61.8	8.4	94 (51; 142) *	20.7	82.9	171 (99; 248) *	13.5	54.0
**_3**	254 (124; 388) *	6.0	59.7	---	47 (−18; 116)	4.3	42.9	203 (92; 319) *	6.5	64.5
**_4**	21 (−49; 94)	1.6	4.7	43.5	12 (−23; 51)	3.3	10.0	10 (−49; 74)	1.0	3.1
**2006_1**	249 (168; 336) *	15.6	62.4	2.4	60 (19; 105) *	14.1	56.3	190 (120; 265) *	16.1	64.5
**_2**	−11 (−76; 60)	−1.0	−3.0	200.9	−27 (−57; 9)	−8.8	−26.3	15 (−42; 75)	1.5	4.6
**_3**	191 (111; 276) *	12.0	48.1	17.9	57 (17; 101) *	13.8	55.2	135 (66; 208) *	11.4	45.7
**_4**	369 (237; 506) *	8.5	93.4	36.7	66 (2; 136) *	5.9	65.3	308 (194; 427) *	9.5	105.0
**2010_1**	211 (133; 293) *	14.4	57.4	1.8	35 (−3; 78)	9.2	36.9	178 (110; 250) *	16.3	65.1
**_2**	101 (10; 197) *	4.6	27.7	1.91	52 (6; 103) *	9.3	55.5	51 (−28; 133)	3.1	18.5
**_3**	471 (354; 593) *	14.4	129.9	5.2	105 (47; 168) *	12.4	111.9	366 (265; 472) *	15.1	136.2
**2013_1**	418 (330; 511) *	24.3	121.7	37.3	96 (54; 143) *	23.0	115.1	325 (248; 407) *	25.0	125.0
**_2**	259 (184; 340) *	19.5	77.8	---	81 (43; 123) *	23.7	94.7	176 (112; 246) *	17.7	70.8
**_3**	116 (15; 222) *	4.3	34.6	33.5	−25 (−73; 29)	−3.6	−29.0	139 (51; 231) *	7.0	55.6
**2015_1**	304 (208; 405) *	13.6	95.0	9.5	91 (43; 144) *	16.1	112.9	217 (134; 304) *	12.9	90.3
**_2**	160 (49; 276) *	5.0	50.3	24.2	12 (−42; 70)	1.6	15.6	148 (52; 249) *	6.2	61.7
**_3**	847 (711; 988) *	20.4	265.4	22.4	105 (42; 174) *	10.2	132.2	750 (630; 874) *	24.1	313.3
**_4**	211 (117; 311) *	9.4	65.8	54.9	55 (9; 105) *	9.7	67.8	158 (76; 245) *	9.3	65.2

**Table 3:** Relationships of cumulative excess mortality for the whole population, the younger (0–64 years) and the elderly (65+ years) age groups to heat wave characteristics (independent variables x), namely cumulative excess heat factor (∑EHF), cumulative significance index (∑EHIsig), cumulative acclimatization index (∑EHIaccl), average daily mean temperature (avgTmean), maximum daily mean temperature (maxTmean), and length of heat waves (duration). Regression coefficients are reported along with their associated R^2^. All relationships were statistically significant at *p* < 0.001.

Variable	Whole Population		0–64 Years		65+ Years	
	Slope	R^2^	Slope	R^2^	Slope	R^2^
∑EHF (°C)	0.81	0.76	0.54	0.38	0.91	0.80
∑EHIsig (°C)	3.97	0.72	2.76	0.39	4.39	0.74
∑EHIaccl (°C)	2.99	0.57	2.33	0.39	3.23	0.56
avgTmean (°C)	31.9	0.31	16.3	0.09	37.5	0.36
maxTmean (°C)	21.2	0.31	11.8	0.11	24.6	0.39
duration (days)	13.9	0.59	11.4	0.45	14.7	0.56

## Supplementary Materials

The following are available online at www.mdpi.com/1660-4601/14/12/1562/s1, Table S1: Characteristics of heat waves during 1994–2015 in the Czech Republic, Table S2A: Impact of heat waves on mortality due to natural causes for the whole population, males and females, and the younger (0–64 years) and the elderly (65+ years) population groups, during 1994–2004 in the Czech Republic, Table S2B: Impact of heat waves on mortality due to natural causes for the whole population, males and females, and the younger (0–64 years) and the elderly (65+ years) population groups, during 2005–2015 in the Czech Republic, Table S3: Net mortality change over extended heat wave periods during 1994–2015 in the Czech Republic, Figure S1: Population structure in the Czech Republic in 1994 and 2015, Figure S2: Top: Daily numbers of natural-cause deaths per standardized 10 million inhabitants in the Czech Republic during 1994–2015. The black line denotes the mortality baseline adjusted for long-term trend, seasonality, weekly cycle, and the effect of heat waves. Bottom: Relative mortality deviations (excess mortality) from the baseline in the same population and period.
