# Supplementary material for: Impacts of the 2015 Heat Waves on Mortality in the Czech Republic—A Comparison with Previous Heat Waves
Source: Int J Environ Res Public Health. 2017 Dec 13;14(12):1562. doi: 10.3390/ijerph14121562 (PMC5750980; doi:10.3390/ijerph14121562)
Supplement: Supplementary file 1 [file ijerph-14-01562-s001.pdf]

Impacts of the 2015 heat waves on mortality in the Czech Republic – A comparison with previous heat waves

Supplementary Material

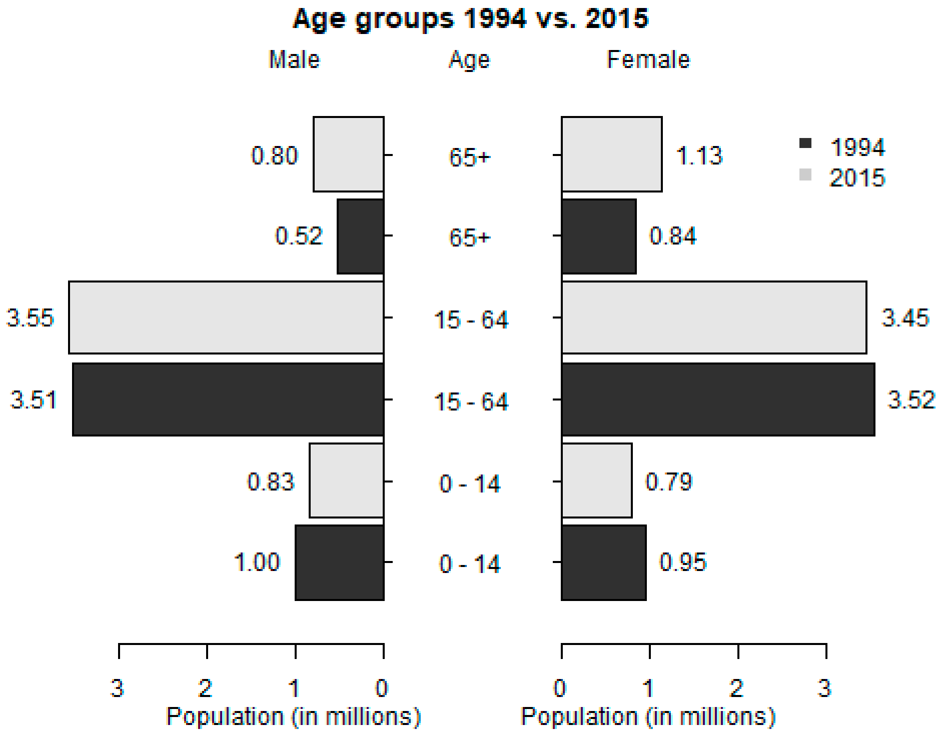

Figure S1. Population structure in the Czech Republic in 1994 and 2015. The data from the Czech Statistical Office are available online at: https://www.czso.cz/staticke/animgraf/cz/.

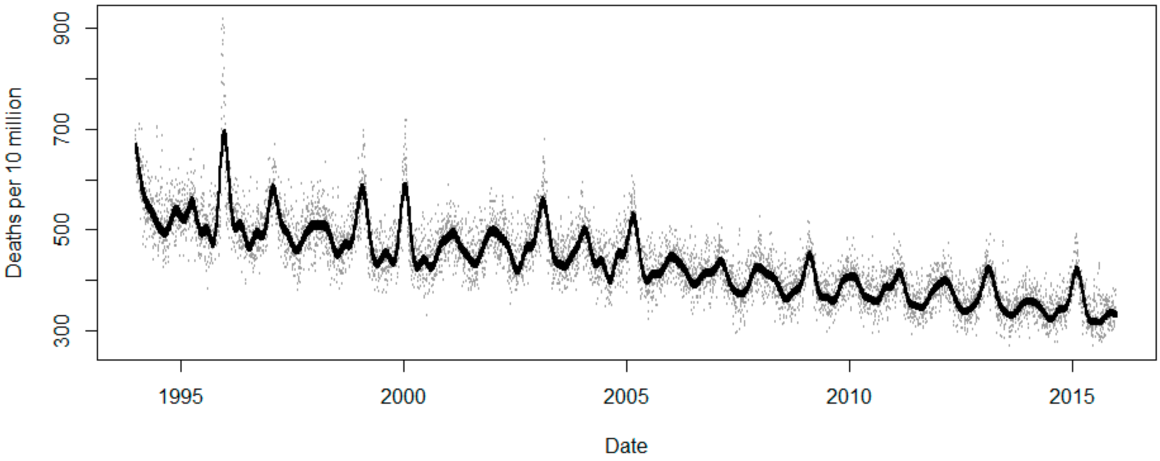

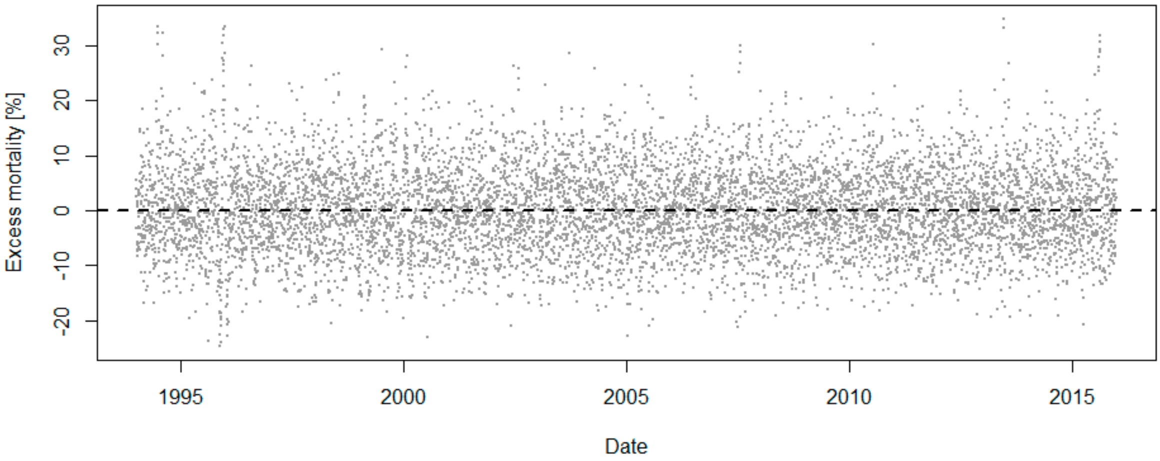

Figure S2 *Top*: Daily numbers of natural-cause deaths per standardized 10 million inhabitants in the Czech Republic during 1994–2015. The black line denotes the mortality baseline adjusted for long-term trend, seasonality, weekly cycle, and the effect of heat waves. *Bottom*: Relative mortality deviations (excess mortality) from the baseline in the same population and period.

Table S1. Characteristics of heat waves during 1994–2015 in the Czech Republic.

Heat wave	Start	End	Days	avgTmean (°C)	maxTmean (°C)	∑EHIsig (°C)	∑EHIaccl (°C)	∑EHF (°C)
1994_1	26–Jun	29–Jun	4	24.9	26.0	9.1	34.9	99.4
_2	2–Jul	4–Jul	3	22.8	24.2	0.7	14.2	10.0
_3	12–Jul	17–Jul	6	22.0	23.2	2.8	15.4	12.2
_4	22–Jul	8–Aug	18	24.1	26.9	51.5	49.3	176.6
1995_1	8–Jul	14–Jul	7	22.8	24.1	8.9	41.7	70.1
_2	20–Jul	22–Jul	3	23.4	24.5	1.7	8.2	11.3
1996_1	7–Jun	10–Jun	4	22.9	23.3	3.0	31.0	35.9
1998_1	5–Jun	7–Jun	3	24.2	25.0	3.5	23.2	37.2
_2	20–Jul	23–Jul	4	24.2	26.5	8.4	26.5	62.8
1999_1	3–Jul	6–Jul	4	23.3	25.1	4.1	25.7	43.1
2000_1	20–Jun	22–Jun	3	24.8	25.5	4.5	18.6	42.9
_2	14–Aug	21–Aug	8	22.7	24.7	8.3	39.3	45.7
2001_1	14–Aug	19–Aug	6	22.2	23.5	0.4	18.6	20.2
2002_1	18–Jun	23–Jun	6	23.2	24.9	9.4	38.3	67.3
_2	8–Jul	10–Jul	3	23.0	24.1	0.3	8.7	8.0
_3	28–Jul	31–Jul	4	22.3	23.9	0.3	11.9	12.1
2003_1	10–Jun	12–Jun	3	23.2	24.3	3.6	16.9	21.1
_2	19–Jul	22–Jul	4	22.8	25.0	3.1	15.2	20.0
_3	1–Aug	10–Aug	10	23.3	25.4	17.1	30.7	72.7
_4	12–Aug	14–Aug	3	23.5	25.8	5.1	4.4	8.2
2004_1	17–Jul	22–Jul	6	22.1	23.3	2.2	33.4	27.1
_2	10–Aug	12–Aug	3	22.6	24.1	0.7	7.8	4.9
_3	17–Aug	19–Aug	3	22.8	23.5	0.7	4.6	5.6
2005_1	28–May	30–May	3	22.9	24.0	2.9	29.7	32.5
_2	27–Jul	30–Jul	4	24.8	27.4	10.0	24.2	77.3
2006_1	19–Jun	22–Jun	4	22.7	23.8	3.3	32.2	34.4
_2	24–Jun	26–Jun	3	22.9	23.7	1.0	19.2	12.7
_3	10–Jul	13–Jul	4	23.1	24.7	6.2	9.6	15.9
_4	18–Jul	28–Jul	11	24.1	25.5	26.5	30.4	99.7
2007_1	14–Jul	21–Jul	8	24.5	27.2	19.8	48.5	171.3
2008_1	26–Jul	1–Aug	7	22.3	23.2	3.4	26.0	23.1
2009_1	14–Jul	17–Jul	4	22.2	23.4	0.6	17.0	8.9
2010_1	9–Jun	12–Jun	4	23.1	24.2	5.8	38.3	59.9
_2	29–Jun	4–Jul	6	22.3	23.5	3.7	29.4	25.4
_3	9–Jul	17–Jul	9	24.5	26.3	24.1	48.3	163.7
2011_1	22–Aug	26–Aug	5	24.2	26.0	10.2	28.6	67.7
2012_1	18–Jun	20–Jun	3	22.8	24.4	4.1	19.8	27.3
_2	29–Jun	2–Jul	4	23.8	26.2	7.6	24.0	55.7
_3	26–Jul	28–Jul	3	22.5	23.3	0.9	8.3	4.6
_4	19–Aug	22–Aug	4	23.6	26.4	6.5	17.1	38.2
2013_1	17–Jun	21–Jun	5	24.6	26.5	13.5	52.8	158.7
_2	26–Jul	29–Jul	4	25.4	28.5	13.2	24.5	90.0
_3	1–Aug	8–Aug	8	24.1	26.7	17.7	22.5	65.2
2014_1	8–Jun	11–Jun	4	24.1	24.9	6.7	35.8	74.4
_2	18–Jul	22–Jul	5	23.0	25.6	8.7	27.3	50.7
2015_1	1–Jul	7–Jul	7	24.4	26.9	16.0	46.3	143.1
_2	16–Jul	25–Jul	10	24.2	27.6	26.0	48.2	145.1
_3	3–Aug	15–Aug	13	26.0	28.3	57.6	55.3	292.4
_4	27–Aug	1–Sep	6	22.5	25.4	8.1	4.9	26.2

Table S2A. Impact of heat waves on mortality for the whole population, males and females, and the younger (0–64 yrs.) and the elderly (65+ yrs.) population groups, during 1994–2004 in the Czech Republic. The variables represent sum of excess deaths per standardized 10,000,000 inhabitants (excess mortality) and its 95% confidence intervals (CI), mean relative mortality deviation (ØRMD), and cumulative relative mortality deviation (∑RMD) during heat waves.

Heat wave	Whole population			Males			Females			0–64 yrs.			65+ yrs.		
	Excess mortality (n)	ØRMD (%)	∑RMD (%)	Excess mortality (n)	ØRMD (%)	∑RMD (%)	Excess mortality (n)	ØRMD (%)	∑RMD (%)	Excess mortality (n)	ØRMD (%)	∑RMD (%)	Excess mortality (n)	ØRMD (%)	∑RMD (%)
1994_1	552 (455;654)	26.8	107.2	325 (249;405)	25.3	101.3	229 (170;294)	29.4	117.4	63 (17;115)	10.9	43.7	487 (403;577)	32.7	130.8
_2	89 (12;170)	5.8	17.3	70 (10;135)	7.4	22.1	19 (−27;69)	3.2	9.6	53 (12;98)	12.3	36.9	36 (−28;105)	3.3	9.8
_3	144 (36;257)	4.8	28.9	79 (−6;168)	4.3	25.5	65 (−1;136)	5.7	34.2	21 (−35;80)	2.4	14.3	123 (32;220)	5.7	34.1
_4	1197 (1002;1397)	13.4	240.3	664 (511;822)	11.8	212.7	534 (414;658)	15.9	285.6	352 (250;459)	14.1	254.4	844 (678;1014)	13.0	234.1
1995_1	512 (391;639)	14.7	102.6	282 (187;381)	13.0	91.0	230 (155;310)	17.3	121.1	114 (52;181)	11.7	82.2	397 (294;505)	15.8	110.4
_2	42 (−34;121)	2.8	8.3	38 (−21;102)	4.1	12.3	4 (−42;53)	0.6	1.7	−13 (−50;30)	−3.0	−9.1	53 (−11;122)	4.9	14.8
1996_1	171 (83;263)	8.9	35.5	74 (6;146)	6.1	24.4	99 (45;157)	13.7	54.7	54 (8;104)	9.8	39.2	117 (44;196)	8.5	33.9
1998_1	231 (154;312)	16.7	50.0	142 (82;207)	16.4	49.1	87 (41;137)	16.7	50.0	27 (−11;69)	7.0	21.0	203 (137;274)	20.2	60.6
_2	373 (284;468)	20.2	80.9	174 (106;248)	15.3	61.0	200 (144;262)	28.6	114.4	94 (48;145)	18.5	73.9	283 (207;365)	21.2	84.7
1999_1	357 (269;450)	20.0	80.1	215 (146;288)	19.6	78.2	143 (89;201)	20.9	83.7	63 (20;112)	12.8	51.1	294 (219;374)	22.9	91.4
2000_1	150 (78;228)	11.4	34.3	76 (20;137)	9.2	27.7	76 (32;126)	15.2	45.6	43 (6;84)	11.9	35.8	108 (47;175)	11.2	33.7
_2	415 (296;540)	12.1	96.4	200 (107;297)	9.3	74.6	215 (141;294)	16.4	131.4	102 (42;168)	10.9	86.9	314 (213;421)	12.5	100.0
2001_1	209 (107;316)	7.9	47.6	90 (12;174)	5.6	33.4	119 (55;187)	11.7	70.1	107 (54;165)	15.2	91.4	104 (18;195)	5.4	32.2
2002_1	418 (313;527)	16.1	96.6	279 (197;366)	17.4	104.6	139 (76;208)	14.0	83.9	102 (50;160)	14.6	87.8	317 (227;411)	16.7	100.1
_2	44 (−26;118)	3.4	10.3	−8 (−60;50)	−0.9	−2.7	51 (8;99)	10.3	31.0	7 (−29;46)	1.9	5.7	38 (−21;102)	4.1	12.3
_3	128 (46;214)	7.5	29.8	72 (8;140)	6.7	26.8	57 (7;112)	8.6	34.4	15 (−26;60)	3.2	12.7	115 (45;190)	9.1	36.4
2003_1	40 (−31;115)	3.0	8.9	3 (−51;62)	0.3	1.0	36 (−8;85)	7.1	21.2	19 (−17;59)	5.4	16.1	21 (−40;85)	2.0	6.1
_2	265 (181;355)	15.5	61.8	183 (116;254)	17.1	68.2	81 (31;136)	12.5	49.9	94 (51;142)	20.7	82.9	171 (99;248)	13.5	54.0
_3	254 (124;388)	6.0	59.7	146 (44;252)	5.5	54.8	108 (30;192)	6.8	67.8	47 (−18;116)	4.3	42.9	203 (92;319)	6.5	64.5
_4	21 (−49;94)	1.6	4.7	−11 (−64;48)	−1.3	−4.0	31 (−12;78)	6.4	19.2	12 (−23;51)	3.3	10.0	10 (−49;74)	1.0	3.1
2004_1	217 (117;322)	8.7	52.3	177 (98;261)	11.4	68.4	41 (−19;105)	4.3	25.7	56 (6;110)	8.3	50.1	160 (75;250)	8.7	52.5
_2	−14 (−80;57)	−1.1	−3.4	−22 (−73;35)	−2.9	−8.6	11 (−30;56)	2.3	6.9	−32 (−63;5)	−9.8	−29.3	19 (−39;80)	2.0	6.0
_3	112 (43;186)	9.2	27.6	65 (11;124)	8.6	25.7	47 (6;94)	10.2	30.5	25 (−10;63)	7.5	22.6	90 (31;154)	9.9	29.8

Table S2B Impact of heat waves on mortality for the whole population, males and females, and the younger (0–64 yrs.) and the elderly (65+ yrs.) population groups, during 2005–2015 in the Czech Republic. The variables represent sum of excess deaths per standardized 10,000,000 inhabitants (excess mortality) and its 95% confidence intervals (CI), mean relative mortality deviation (ØRMD), and cumulative relative mortality deviation (∑RMD) during heat waves.

Heat wave	Whole population			Males			Females			0–64 yrs.			65+ yrs.		
	Excess mortality (n)	ØRMD (%)	∑RMD (%)	Excess mortality (n)	ØRMD (%)	∑RMD (%)	Excess mortality (n)	ØRMD (%)	∑RMD (%)	Excess mortality (n)	ØRMD (%)	∑RMD (%)	Excess mortality (n)	ØRMD (%)	∑RMD (%)
2005_1	85 (16;159)	7.0	20.9	36 (−18;94)	4.7	14.0	50 (8;97)	10.7	32.2	28 (−7;67)	8.9	26.7	60 (2;124)	6.6	19.7
_2	265 (182;353)	16.1	64.5	175 (110;245)	17.3	69.3	90 (40;146)	14.2	56.9	10 (−30;54)	2.1	8.6	255 (182;332)	21.1	84.4
2006_1	249 (168;336)	15.6	62.4	174 (110;243)	17.6	70.5	77 (28;130)	12.5	49.8	60 (19;105)	14.1	56.3	190 (120;265)	16.1	64.5
_2	−11 (−76;60)	−1.0	−3.0	−5 (−56;51)	−0.8	−2.5	−7 (−46;38)	−1.4	−4.3	−27 (−57;9)	−8.8	−26.3	15 (−42;75)	1.5	4.6
_3	191 (111;276)	12.0	48.1	136 (74;204)	14.1	56.2	55 (7;108)	8.9	35.6	57 (17;101)	13.8	55.2	135 (66;208)	11.4	45.7
_4	369 (237;506)	8.5	93.4	161 (59;267)	6.0	66.4	213 (130;301)	12.6	138.4	66 (2;136)	5.9	65.3	308 (194;427)	9.5	105.0
2007_1	628 (512;748)	20.9	167.5	445 (354;542)	24.2	193.4	182 (113;256)	15.7	125.7	84 (28;144)	10.5	84.3	544 (443;649)	24.6	197.1
2008_1	296 (194;404)	11.6	81.5	140 (61;224)	8.8	61.9	159 (96;227)	16.4	114.8	106 (53;163)	15.6	109.5	192 (105;284)	10.2	71.6
2009_1	28 (−46;107)	1.9	7.6	22 (−36;84)	2.4	9.5	7 (−38;56)	1.2	4.6	−24 (−58;16)	−6.2	−24.8	52 (−13;121)	4.7	18.9
2010_1	211 (133;293)	14.4	57.4	115 (54;180)	12.5	49.9	96 (49;149)	17.4	69.5	35 (−3;78)	9.2	36.9	178 (110;250)	16.3	65.1
_2	101 (10;197)	4.6	27.7	22 (−49;98)	1.6	9.6	80 (24;141)	9.9	59.3	52 (6;103)	9.3	55.5	51 (−28;133)	3.1	18.5
_3	471 (354;593)	14.4	129.9	238 (147;335)	11.6	104.1	234 (162;311)	19.4	174.3	105 (47;168)	12.4	111.9	366 (265;472)	15.1	136.2
2011_1	207 (123;296)	11.8	59.0	115 (49;186)	10.5	52.3	92 (41;148)	14.0	70.1	51 (10;97)	11.5	57.3	157 (85;235)	12.0	59.9
2012_1	45 (−18;113)	4.2	12.7	25 (−25;78)	3.7	11.2	20 (−18;63)	5.0	14.9	13 (−18;48)	4.8	14.3	34 (−21;92)	4.2	12.5
_2	143 (69;222)	10.5	41.9	63 (6;125)	7.4	29.7	80 (35;131)	15.4	61.5	31 (−6;72)	9.0	35.9	114 (50;183)	11.2	44.7
_3	128 (64;197)	12.5	37.6	101 (50;156)	15.9	47.8	27 (−12;69)	6.8	20.3	20 (−11;56)	7.5	22.4	108 (53;168)	14.2	42.6
_4	39 (−33;114)	2.7	10.8	25 (−31;85)	2.7	10.9	12 (−31;60)	2.3	9.0	−38 (−71;−1)	−10.8	−43.2	75 (13;142)	7.2	28.9
2013_1	418 (330;511)	24.3	121.7	214 (147;287)	20.2	101.0	206 (151;266)	31.3	156.6	96 (54;143)	23.0	115.1	325 (248;407)	25.0	125.0
_2	259 (184;340)	19.5	77.8	177 (118;242)	21.5	85.9	82 (37;132)	16.0	64.0	81 (43;123)	23.7	94.7	176 (112;246)	17.7	70.8
_3	116 (15;222)	4.3	34.6	13 (−65;95)	0.7	5.7	103 (40;171)	10.1	81.0	−25 (−73;29)	−3.6	−29.0	139 (51;231)	7.0	55.6
2014_1	196 (122;275)	14.8	59.2	158 (99;222)	19.2	76.6	39 (−5;87)	7.8	31.0	45 (10;85)	13.5	54.2	152 (88;220)	15.2	61.0
_2	161 (80;246)	9.9	49.3	165 (100;234)	16.4	82.0	−4 (−50;48)	−0.6	−3.2	36 (−3;80)	9.1	45.5	123 (53;197)	10.0	49.8
2015_1	304 (208;405)	13.6	95.0	222 (146;303)	16.1	112.6	82 (24;144)	9.5	66.3	91 (43;144)	16.1	112.9	217 (134;304)	12.9	90.3
_2	160 (49;276)	5.0	50.3	60 (−26;151)	3.1	30.7	102 (33;176)	8.4	83.5	12 (−42;70)	1.6	15.6	148 (52;249)	6.2	61.7
_3	847 (711;988)	20.4	265.4	478 (373;589)	18.8	245.0	371 (286;461)	23.1	300.1	105 (42;174)	10.2	132.2	750 (630;874)	24.1	313.3
_4	211 (117;311)	9.4	65.8	114 (46;187)	9.6	57.5	68 (11;131)	7.9	55.0	55 (9;105)	9.7	67.8	158 (76;245)	9.3	65.2

Table S3. Sum of excess deaths per standardized 10,000,000 inhabitants during the extended heat wave periods (EHPs) during 1994–2015. Duration denotes the length of EHP. Displaced mortality represents the absolute value of the ratio of the sum of three-day-averaged mortality deviations during the negative phase of EHP (see section 2.5.3 in the main manuscript) to the sum of three-day-averaged mortality deviations during the positive phase. --- indicates that the heat wave was considered as a single EHP together with the following one.

Heat wave	Duration (days)	Excess mortality (n)	Displaced mortality (%)
1994_1	---	---	---
_2	26	821 (589;1056)	18.4
_3	10	71 (−69;214)	28.3
_4	37	888 (617;1163)	26.6
1995_1	20	749 (548;954)	2.4
_2	10	−171 (−306;−33)	775.5
1996_1	8	213 (89;341)	5.0
1998_1	26	374 (155;596)	30.5
_2	16	231 (62;404)	43.9
1999_1	19	622 (437;810)	6.3
2000_1	11	−46 (−180;92)	123.4
_2	25	312 (107;521)	36.5
2001_1	13	88 (−60;240)	61.7
2002_1	14	371 (216;530)	12.6
_2	12	185 (44;330)	1.4
_3	12	291 (148;438)	13.5
2003_1	5	48 (−45;143)	21.8
_2	20	562 (377;752)	8.4
_3	---	---	---
_4	17	156 (−12;328)	43.5
2004_1	16	281 (120;447)	22.3
_2	15	34 (−119;189)	199.8
_3	5	23 (−65;114)	111.1
2005_1	13	189 (44;337)	10.0
_2	8	274 (159;394)	5.8
2006_1	16	562 (400;727)	2.4
_2	11	−33 (−161;99)	200.9
_3	12	224 (88;364)	17.9
_4	30	296 (81;514)	36.7
2007_1	29	441 (234;651)	38.4
2008_1	13	285 (147;426)	18.9
2009_1	8	51 (−55;161)	27.7
2010_1	9	288 (172;408)	1.8
_2	9	103 (−10;219)	1.9
_3	14	414 (271;562)	5.2
2011_1	18	−12 (−166;146)	112.4
2012_1	15	136 (−7;282)	29.8
_2	13	243 (110;380)	18.6
_3	16	355 (207;507)	3.1
_4	10	36 (−79;154)	29.6
2013_1	18	299 (143;459)	37.3
_2	---	---	---
_3	29	363 (168;561)	33.5
2014_1	20	102 (−58;264)	69.1
_2	18	316 (163;472)	4.0
2015_1	16	397 (253;545)	9.5
_2	15	120 (−17;259)	24.2
_3	23	681 (506;859)	22.4
_4	16	92 (−49;237)	54.9
